# Two years' experience with bicaval dual lumen cannula for venovenous extracorporeal membrane oxygenation in adult refractory acute respiratory distress syndrome

**DOI:** 10.1186/cc10706

**Published:** 2012-03-20

**Authors:** J Reeb, PE Falcoz, J Pottecher, X Delabranche, N Santelmo, A Steib, M Hasselmann, G Massard

**Affiliations:** 1 Hôpitaux universitaires de Strasbourg, France

## Introduction

Venovenous extracorporeal membrane oxygenation (ECMO) is a respiratory support increasingly used in adult refractory acute respiratory distress syndrome (ARDS). Technological advances such as bicaval dual lumen cannula (DLC) allow one to decrease drawbacks associated with this cardiopulmonary bypass technique and to implement it in the ICU setting. We report our 2 years' experience of using DLC for ECMO in adult refractory ARDS.

## Methods

A prospective single-center study between November 2009 and November 2011 including all medical and surgical adult patients receiving ECMO for refractory ARDS. All ECMOs were performed with DLC implanted percutaneously in the right internal jugular vein. Variables under study were: arterial blood gases, duration of ECMO support, activated cephalin time (TCA) values, number of blood products transfused, and patient's outcome. Statistical test: Student's *t *test.

## Results

Twenty-five ECMOs were performed in 24 patients (16 men and eight women). Mean age of patients was 52.2 years ± 17.5. All these patients had severe ARDS despite optimal medical therapy. At DLC implantation, mean pH, PaCO_2_, PaO_2_, and PaO_2_/FiO_2 _ratio were 7.25 ± 0.11, 60.5 ± 17.5 mmHg, 58.9 ± 13.6 mmHg, and 61 ± 14 respectively. Mean duration of respiratory support with ECMO was 9.5 ± 4.8 days and mean blood flow was 3.3 ± 0.6 l/minute. During ECMO, arterial blood gases were significantly improved (*P *< 0.05): mean PaCO_2_, and PaO_2 _were 39.9 ± 4.8 mmHg, and 92.7 ± 21.1 mmHg respectively. Concerning haemostasis and provision of blood products, mean TCA and mean pellets of red blood cells transfused were 53.6 ± 1.2 seconds and 10.7 ± 7 respectively. Eleven patients (46%) died under ECMO. Causes of death with ECMO support were: five multiorgan failures, two septic shocks, two withdrawal of care, one hemodynamic shock, and one refractory hypoxemia. ECMO withdrawal was possible in 13 patients (twice in one patient) with PaO_2_/FiO_2 _ratio 258 ± 24 at withdrawal. Removal of the endotracheal tube was performed for eight patients (33%), 18.3 ± 6.2 days after DLC implantation. Eight patients (33%) were discharged alive from the ICU, 18.3 ± 6.3 days after DLC implantation. These eight patients were discharged alive from hospital. No adverse event related to the DLC was observed.

## Conclusion

ECMO with a bicaval DLC is feasible in the ICU. It improves significantly haemostasis parameters in patients suffering from refractory ARDS. DLC also decreases drawbacks associated with the ECMO respiratory support.

